# Supplementary material for: Global Prevalence of Colistin Resistance in Klebsiella pneumoniae from Bloodstream Infection: A Systematic Review and Meta-Analysis
Source: Pathogens. 2022 Sep 24;11(10):1092. doi: 10.3390/pathogens11101092 (PMC9607870; doi:10.3390/pathogens11101092)
Supplement: Supplementary file 1 [file pathogens-11-01092-s001.zip › Table S1 Basic characteristics and quality score of included studies.pdf]

Table S1. Basic characteristics and quality score of included studies.

Study ID	Time of Study	Year of Publication	Country	N. Isolate Bacteria	Quality Score Based on JBI Criteria
Bir et al [23]	2018–2019	2022	India	48	7
Imtiaz et al [24]	2021	2021	Pakistan	31	7
Xiao et al [25]	2016–2019	2021	China	78	8
Naomi-Matsuoka et al [26]	2018	2020	Peru	36	5
Santimaleeworagun et al [27]	2017–2018	2020	Thailand	26	6
Boszczowski et al [28]	2010–2015	2019	Brazil	16	5
Zhang et al [29]	2015–2016	2019	China	241	8
Dong et al [30]	2011–2014	2018	China	164	6
Lee et al [32]	2016–2017	2018	South Korea	597	7
Gandra et al [33]	2008–2014	2016	India	1481	8

References

23. Bir, R.; Gautam, H.; Arif, N.; Chakravarti, P.; Verma, J.; Banerjee, S.; Tyagi, S.; Mohapatra, S.; Sood, S.; Dhawan, B.; et al. Analysis of colistin resistance in carbapenem-resistant *Enterobacterales* and XDR *Klebsiella pneumoniae*. *Ther. Adv. Infect. Dis.* **2022**, *9*, 20499361221080650. https://doi.org/10.1177/20499361221080650.
24. Imtiaz, W.; Syed, Z.; Rafaque, Z.; Andrews, S.C.; Dasti, J.I. Analysis of Antibiotic Resistance and Virulence Traits (Genetic and Phenotypic) in *Klebsiella pneumoniae* Clinical Isolates from Pakistan: Identification of Significant Levels of Carbapenem and Colistin Resistance. *Infect. Drug Resist.* **2021**, *14*, 227–236. https://doi.org/10.2147/idr.s293290.
25. Xiao, S.; Chen, T.; Wang, H.; Zeng, Q.; Chen, Q.; Yang, Z.; Han, L.; Chen, E. Drug Susceptibility and Molecular Epidemiology of *Klebsiella pneumoniae* Bloodstream Infection in ICU Patients in Shanghai, China. *Front. Med.* **2021**, *8*, 754944. https://doi.org/10.3389/fmed.2021.754944.
26. Naomi-Matsuoka, A.; Vargas, M.; Ymaña, B.; Soza, G.; Pons, M.J. Colistin resistance in multidrug-resistant *Klebsiella pneumoniae* strains at a perinatal maternal institute in Lima, Peru, 2015–2018. *Rev. Peru. Med. Exp. Salud Pública* **2020**, *37*, 716–720.
27. Santimaleeworagun, W.; Thunyaharn, S.; Juntanawiwat, P.; Thongnoy, N.; Harindhanavudhi, S.; Nakeesathit, S.; Teschumroon, S. The prevalence of colistin-resistant gram-negative bacteria isolated from hospitalized patients with bacteremia. *J. Appl. Pharm. Sci.* **2020**, *10*, 56–59.
28. Boszczowski, I.; Salomão, M.C.; Moura, M.L.; Freire, M.P.; Guimarães, T.; Cury, A.P.; Rossi, F.; Rizek, C.F.; Martins, R.C.R.; Costa, S.F. Multidrug-resistant *Klebsiella pneumoniae*: Genetic diversity, mechanisms of resistance to polymyxins and clinical outcomes in a tertiary teaching hospital in Brazil. *Rev. Inst. Med. Trop. Sao Paulo* **2019**, *61*, e29. https://doi.org/10.1590/s1678-9946201961029.
29. Zhang, F.; Li, Y.; Lv, Y.; Zheng, B.; Xue, F. Bacterial susceptibility in bloodstream infections: Results from China Antimicrobial Resistance Surveillance Trial (CARST) Program, 2015–2016. *J. Glob. Antimicrob. Resist.* **2019**, *17*, 276–282. https://doi.org/10.1016/j.jgar.2018.12.016.
30. Dong, F.; Zhang, Y.; Yao, K.; Lu, J.; Guo, L.; Lyu, S.; Yang, Y.; Wang, Y.; Zheng, H.; Song, W.; et al. Epidemiology of Carbapenem-Resistant*Klebsiella pneumoniae*Bloodstream Infections in a Chinese Children's Hospital: Predominance of New Delhi Metallo-β-Lactamase-1. *Microb. Drug Resist.* **2018**, *24*, 154–160. https://doi.org/10.1089/mdr.2017.0031.
31. Yong, D.; Shin, H.B.; Kim, Y.K.; Cho, J.; Lee, W.G.; Ha, G.Y.; Choi, T.Y.; Jeong, S.H.; Lee, K.; Chong, Y.; KONSAR group. Increase in the Prevalence of Carbapenem-Resistant Acinetobacter Isolates and Ampicillin-Resistant Non-Typhoidal Salmonella Species in Korea: A KONSAR Study Conducted in 2011. *Infect. Chemother.* **2014**, *46*, 84–93.
32. Lee, H.; Yoon, E.-J.; Kim, D.; Jeong, S.H.; Won, E.J.; Shin, J.H.; Kim, S.H.; Shin, J.H.; Shin, K.S.; Kim, Y.A.; et al. Antimicrobial resistance of major clinical pathogens in South Korea, May 2016 to April 2017: First one-year report from Kor-GLASS. *Eurosurveillance* **2018**, *23*, 1800047. https://doi.org/10.2807/1560-7917.es.2018.23.42.1800047.
33. Nwabor, O.; Terbtothakun, P.; Voravuthikunchai, S.; Chusri, S. A Bibliometric Meta-Analysis of Colistin Resistance in *Klebsiella pneumoniae*. *Diseases* **2021**, *9*, 44. https://doi.org/10.3390/diseases9020044.
